# Elevated fasting serum glucose levels increase the risk of hepatocellular carcinoma: A prospective cohort study

**DOI:** 10.1097/MD.0000000000016369

**Published:** 2019-07-26

**Authors:** Tong Liu, Wanchao Wang, Haozhe Cui, Miaomiao Sun, Yiming Wang, Xining Liu, Liying Cao, Hai Liu, Siqing Liu

**Affiliations:** a Department of Hepatobiliary Surgery, Kailuan General Hospital Affiliated to North China University of Science and Technology; b Department of Graduate School, North China University of Science and Technology; c Department of Anesthesiology, Kailuan General Hospital Affiliated to North China University of Science and Technology, Tangshan, Hebei, China.

**Keywords:** competing risk models, fasting blood glucose, hepatocellular carcinoma, incidence

## Abstract

Previous studies have demonstrated a positive relationship between liver cancer and diabetes mellitus. However, elevated fasting blood glucose (FBG) itself may be a risk factor for the development of hepatocellular carcinoma (HCC) rather than diabetes, and during the follow-up period, death is an event that may occur before the occurrence of HCC, which should be dealt with competing risk models. Our study aims to investigate the relationship between FBG and new-onset HCC by using competing risk regression models.

We prospectively studied the relationship between FBG concentrations and risk of HCC in a cohort of 93,447 participants who were free of prior HCC, and whose demographic characteristics and biochemical parameters were recorded. Cox proportional hazards regression models and competing risk regression models were used to evaluate the association between FBG concentrations and risk of incident HCC.

A total of 302 participants were diagnosed with HCC among 93,447 subjects during 810,499 person-years of follow-up. The multivariable hazard ratios (HRs) [95% confidence interval (95% CI)] for the association of FBG and log(FBG) with HCC were 1.07 (1.01∼1.12), 1.84 (1.23∼2.74) in an analysis adjusted for other potential variables. In the multivariable adjusted analysis, participants who were in 4.82 mmol/L≤FBG≤5.49 mmol/L group and FBG >5.49 mmol/L group would have increased the risk of HCC by 47% and 69%, respectively. In a cause-specific hazard model (CS model), the multivariable HRs (95% CI) for the association of FBG with HCC were 1.46 (1.09∼1.98), 1.69 (1.27∼2.27) in the multivariable adjusted analysis. Similar results were also observed in sub-distribution hazard function model (SD model) with corresponding multivariate HRs (95% CI) of 1.46 (1.09∼2.00), 1.69 (1.25∼2.27) in 4.82 mmol/L≤FBG≤5.49 mmol/L group and FBG >5.49 mmol/L group, respectively.

Higher FBG concentrations itself were positively associated with new-onset HCC in the Cox proportional hazards regression models and competing risk models. FBG concentrations can be used as a scientific and important way to identify individuals with a higher risk of HCC and control of FBG concentrations might serve as a possible way to decrease the risk of HCC among Chinese population.

**Trial registration:** ChiCTR–TNRC–11001489. Registered August 24, 2011 (retrospectively registered).

## Abstract 2

Supplemental Digital Content is available in the text

## 1 Introduction

Hepatocellular carcinoma (HCC) composes 80% to 90% of liver cancer, which represents 6% and 9% of the global cancer incidence and mortality burden.^[1]^ Approximately 85% of all liver cancer cases occurred in Asia and Africa with more than half of the incidence and mortality in China.^[2]^ In Egypt, Japan, and China, incidence rates are more than 20 per 100,000 people. The lowest rates of HCC are those reported for North America, South America, and Europe, which have incidence rates of less than 10 per 100,1000 people.^[2]^ Differences in incidence rates of HCC reflect the various distribution of predisposing conditions. In North and South America, Europe, people with hepatitis C virus (HCV)-infection and nonalcoholic fatty liver disease have experienced increasing hepatocellular carcinoma incidence.^[3]^ On the contrary, diminished aflatoxin exposure is leading to a decline of HCC incidence rates in Asia where hepatitis B virus (HBV)-infection remains a major predisposing condition.^[2]^

Cirrhosis, HBV infections, and HCV infections are established risk factors for the development of HCC.^[4,5]^ Diabetes mellitus has been suggested as a potential risk factor for HCC. The relationship between diabetes mellitus and HCC was first reported in 1986 by Lawson et al.^[6]^ The majority of following studies including meta-analyses and systematic reviews showed strong demonstrations that liver cancer and diabetes mellitus are significantly associated with a few exceptions.^[7–10]^ However, there are several disadvantages in former literature. First, people who suffer from increasing risks of HCC may be free of diabetes but are afflicted with elevated fasting blood glucose (FBG, in mmol/L). Elevated FBG itself may be a risk factor for the development of HCC rather than diabetes. Second, during the follow-up period, death is an event that may occur before the occurrence of HCC, which should be dealt with competing risk regression models. To our knowledge, there are few, if any, studies concerning the effects of elevated FBG levels on the development of HCC. Thus, our study aims to investigate the relationship between FBG and new-onset HCC by using competing risk regression models based on Kailuan Study (Trial identification: ChiCTR–TNRC–11001489; Registration number: 11001489).

## 2 Materials and methods

### 2.1 Kailuan study

Kailuan Study, a prospective population-based study in Kailuan community, is owned and managed by Kailuan Group in Tangshan city in northern China. The study represented the Chinese population from a socioeconomic perspective and was designed to investigate risk factors for chronic diseases.^[11]^

### 2.2 Study population

From July 2006 to October 2007, a total of 101,510 working and retired employees aged 18 to 98 years from Kailuan Corporation underwent physical examinations (the baseline examination) at Kailuan General Hospital and its 10 affiliated hospitals. Information, including physical examinations, Type-B ultrasonic examinations, blood, urine, and biochemical tests were collected. Participants were then followed biennially with repeated questionnaires and medical examinations.

In the current study, we excluded 543 subjects who had a history of cancer at baseline, 3712 subjects with missing data of FBG, and 3808 subjects without measurements of other potential risk factors for HCC, including age, gender, body mass index (BMI, in kg/m^2^), alanine aminotransferase (ALT, in mmol/L), cirrhosis, physical activity, drinking status, smoking status, HBV infection, nonalcoholic steatohepatitis (NASH) or nonalcoholic fatty liver disease (NAFLD), alcoholic liver disease. A total of 93,447 participants were finally recruited in the present study. Considering that elevated FBG, rather than diabetes, may be a risk factor for new-onset HCC, participants were categorized into 3 groups based on FBG tertiles rather than the definition of diabetes mellitus. This study was approved by Ethics Committee of Kailuan General Hospital, and it was in compliance with the Declaration of Helsinki.

### 2.3 Laboratory assessment

Blood samples were obtained from the antecubital veins and transfused into vacuum tubes containing EDTA in the morning after an overnight fasting period. Within 30 minutes of collection, the blood was centrifuged for 10 minutes at 3000 rotations per minute at 25°C. Plasma was separated and stored at -80°C for subsequent analyses. All the plasma samples were analyzed using an auto-analyzer (Hitachi; Hitachi, Tokyo, Japan) at the central laboratory of the Kailuan General Hospital.^[12]^ Fasting blood glucose was measured with the hexokinase/glucose-6-phosphate dehydrogenase method with an upper limit of detection of 30.07 mmol/L. ALT (ALT, in U/L) was measured with an enzymatic rate method with an upper limit of detection of 1000 U/L. Total cholesterol and triglyceride were both measured using enzymatic colorimetric method with an upper limit of detection of 20.68 and 11.30 mmol/L; High-density lipoprotein cholesterol (HDL-C) and low-density lipoprotein cholesterol (LDL-C) were measured by direct test method with respective upper detecting limit of 12.90 and 3.88 mmol/L. The inter-assay coefficient of variation for each measurement was less than 10%. Diabetes was defined as follows: FBG ≥7.0 mmol/L and/or validated physician diagnosis and/or had undergone or was undergoing hypoglycemic therapy.

### 2.4 Questionnaire assessment

Questionnaires were done via face-to-face interviews by the medical staff and trained research nurses. Information on age, sex, socioeconomic status, lifestyle behaviors, and medical history was collected at baseline. Smoking was defined as having smoked at least 1 cigarette per day on average for at least 1 year. Drinking status was defined as having taken alcohol of 100 mL/day (alcohol contents >50%) of alcohol for more than 1 year. Exercise was defined as taking exercises more than 3 times weekly with each time lasting at least 30 minutes.^[11]^

### 2.5 Anthropometric measurements and blood pressure measurement

Details of the collection of anthropometric indices, including height, weight and waist circumference (WC, in cm), and blood pressure were published previously. ^[11]^ BMI was calculated as body weight (kg) divided by the square of height (m^2^). Hypertension was defined as having a history of hypertension, systolic blood pressure ≥140 mm Hg and/or diastolic blood pressure ≥90 mm Hg, or using antihypertensive medications.

### 2.6 Type-B ultrasonic examination and assessment of liver disease

All subjects were required to fasting before examination, and a panel of specialists examined the abdominal region (liver, gallbladder, pancreas, and spleen in turn) of each participant, diagnosing liver disease based on real-time ultrasound sonography (PHILIPS HD-15) with 3.5 MHz. Fatty liver was diagnosed and graded as mild, moderate, and severe according to ultrasonographic liver features by referring to established criteria.^[13]^ Cirrhosis was diagnosed and graded as earlier period cirrhosis and advanced cirrhosis based on ultrasonographic liver features according to established criteria.^[14]^

### 2.7 Outcome ascertainment

Participants were followed from the ending point of the first-time examination till the diagnosis of new-onset HCC, censoring, death, or end of follow-ups (December 31, 2015), whichever event came first. All cancer events were coded using the ICD-10 system to indicate cancer type. Cancer cases in the cohort were confirmed via biennially follow-up examinations with repeated questionnaires and medical examinations. Further outcome information was confirmed by checking discharge summaries from the 11 affiliated hospitals where participants were treated and diagnosed, as well as by evaluating medical records from medical insurance to double-check diagnoses that may have been missed. For the participants without face-to-face follow-up, the outcome information was obtained directly by checking death certificates from the provincial vital statistics offices, discharge summaries, and medical records.^[15]^

### 2.8 Statistical analysis

Data input was carried out by trained personnel of each participating hospital. All statistical analyses were performed using SAS software, version 9.4. Variables that were normally distributed were presented as mean (standard deviation), and compared using 1-way analysis of variance (ANOVA). Data in the skewed distribution were described by median (interquartile range) and analyzed by the nonparametric tests. Categorical variables were described by percentage and compared using the Chi-square test. Logarithmic transformation was used for baseline FBG for analyses with continuous variables to decrease the effect of extreme observations. Cox proportional hazard models adjusted for suspected confounders were used to calculate hazard ratios (HRs) and 95% confidence intervals (95% CIs) for baseline FBG and new-onset HCC, with adjustments for age, sex, BMI, ALT, cirrhosis (yes/no), HBV infection (positive/negative), NASH/NAFLD (yes/no), alcoholic liver disease (yes/no), current smoker (yes/no), drinking status (yes/no), and physical activity (yes/no). The dose--response association was calculated by restricted cubic spline regression (RCS). Similar analytic methods were used to test the effects of 3 pre-specified FBG groups on the risk of HCC. During the long period of follow-up, death may occur before the occurrence of HCC, traditional multivariate COX regression model may substantially overestimate the absolute risk of the event of interest. In that case, cause-specific hazard model and sub-distribution hazard function model were used to calculate the absolute risk of HCC. As a sensitivity analysis, we further excluded 8456 participants who suffered from diabetes and 32 participants who occurred HCC within 1 year entering to the cohort. Reported *P* values are 2-sided, and *P* < .05 was recorded as a significant difference.

## 3 Results

### 3.1 General characteristics

The baseline characteristics for participants stratified by FBG tertiles are summarized in Table 1. Compared with the lower FBG concentrations, the participants in the higher FBG concentrations were older in age, and higher in SBP, DBP, WC, BMI, TC, TG, LDL, ALT, and lower in HDL. Higher FBG concentrations were also associated with higher percentage of males, physical activity, alcohol drinking, smoking, hypertension, NASH/NALFD, and alcoholic liver disease. There was no difference in the prevalence of cirrhosis among 3 groups.

**Table 1:**
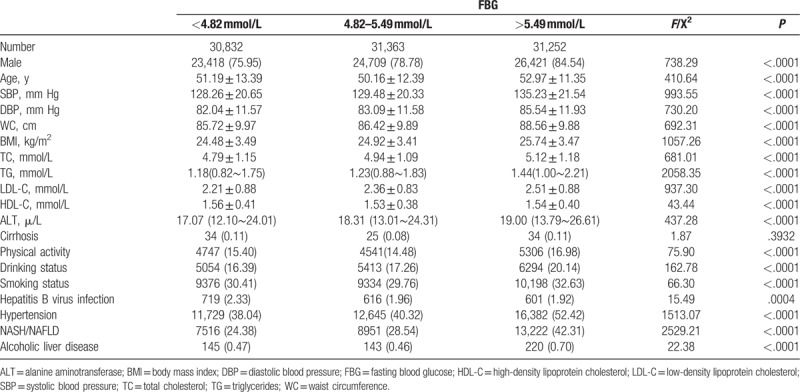
Baseline characteristics of the participants stratified by FBG subgroups.

### 3.2 Incidence of HCC

The total follow-up time was 810,499 person-years, with a mean follow-up time of 8.67 ± 1.36 years per participant. A total of 302 participants were identified to have newly diagnosed HCC among 93,447 subjects. The mean age was 51.44 ± 12.45 with 74,548 (79.78%) males and 18,899 (20.22%) females in our study. The crude incidence of HCC per 10,000 person-years was 3.73 in all participants (1.15 per 10,000 person-years for women, 4.39 per 10,000 person-years for men). Our study indicated a clear trend based on FBG concentrations, where age- and sex-standardized incidence of HCC monotonically increased from 2.05 per 10,000 person-years to 3.10 per 10,000 person-years and 4.10 per 10,000 person-years in each group of FBG <4.82 mmol/L, 4.82 mmol/L≤FBG≤5.49 mmol/L, and FBG >5.49 mmol/L, respectively.

### 3.3 The association between FBG levels and HCC risk

**Figure 1:**
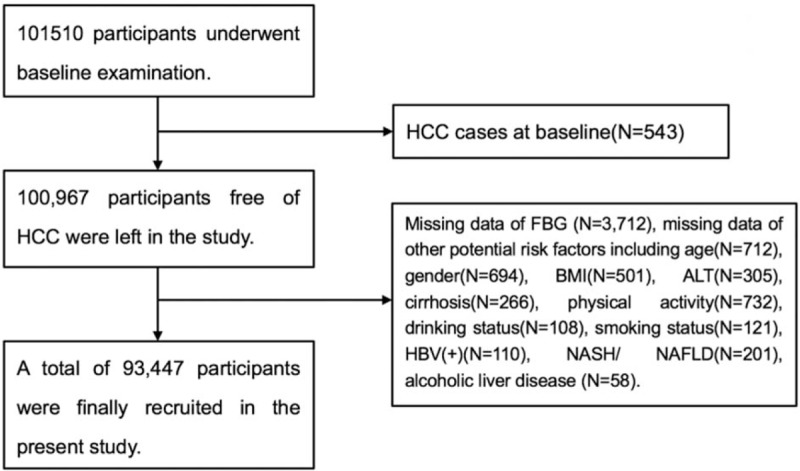
The procedure of participants screening.

Table 2 displays the crude and adjusted HRs (95% CI) for newly diagnosed HCC events. The HRs showed the effect on HCC risk per unit of FBG and log(FBG). In the univariate analysis, the HRs (95% CI) for the association of FBG and log(FBG) with new-onset HCC were 1.10 (1.04∼1.15) and 2.22 (1.47∼3.35), respectively. The multivariable HRs (95% CI) for the association of FBG and log(FBG) with HCC were 1.07 (1.01∼1.12), 1.84 (1.23∼2.74) in an analysis that included age, sex, BMI, ALT, cirrhosis, HBV infection, NASH/NAFLD, alcoholic liver disease, current smoker, drinking status, and physical activity. The RCS model showed a positive dose--response but nonlinear association between FBG levels and the risk of HCC among the participants (*P*-overall = .0037, *P*-nonlinear = .0410; Fig. 1). Statistically significant associations were also observed for the associations of tertiles of FBG with HCC in the univariate analysis with the corresponding HRs (95% CI) of 1.31 (0.97∼1.76), 1.66 (1.25∼2.21) in 4.82 mmol/L≤FBG≤5.49 mmol/L group and FBG >5.49 mmol/L group, respectively (Table 3). After adjusting for other aforementioned confounding factors, the association between tertiles of FBG and HCC was attenuated but remained significant with the corresponding HRs (95%CI) of 1.47 (1.09∼1.98), 1.69 (1.27∼2.27) in 4.82 mmol/L≤FBG≤5.49 mmol/L group and FBG >5.49 mmol/L group, respectively (Table 3).

**Table 2:**
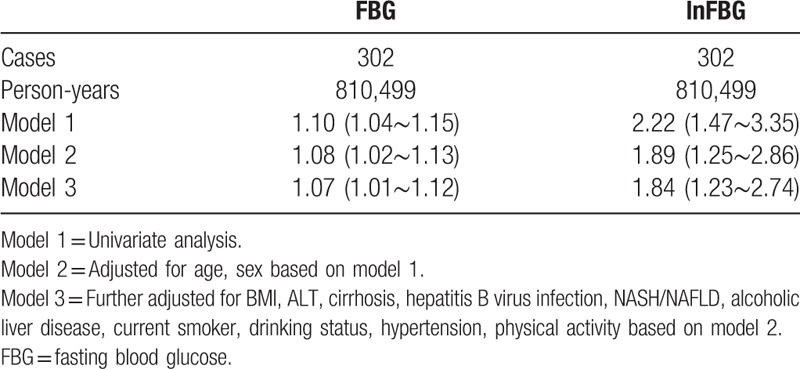
Hazard ratios and 95% confidence interval (CI) of FBG level for risk of HCC.

**Table 3:**
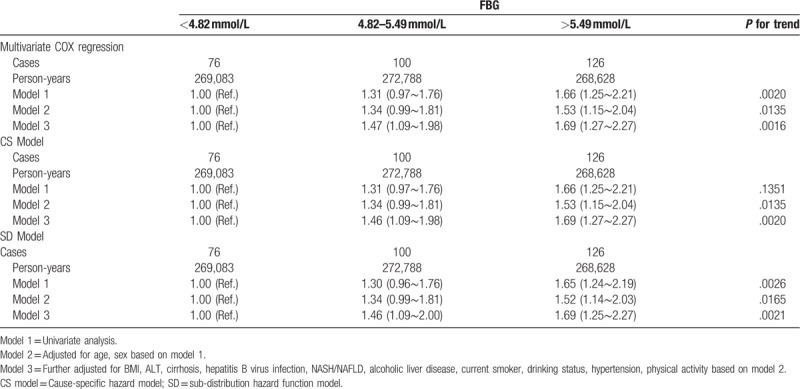
Hazard ratios and 95% confidence interval (CI) for risk of HCC among participants stratified by FBG subgroups in different regression models.

### 3.4 The association between FBG levels and HCC risk in competing risk regression model

**Figure 2:**
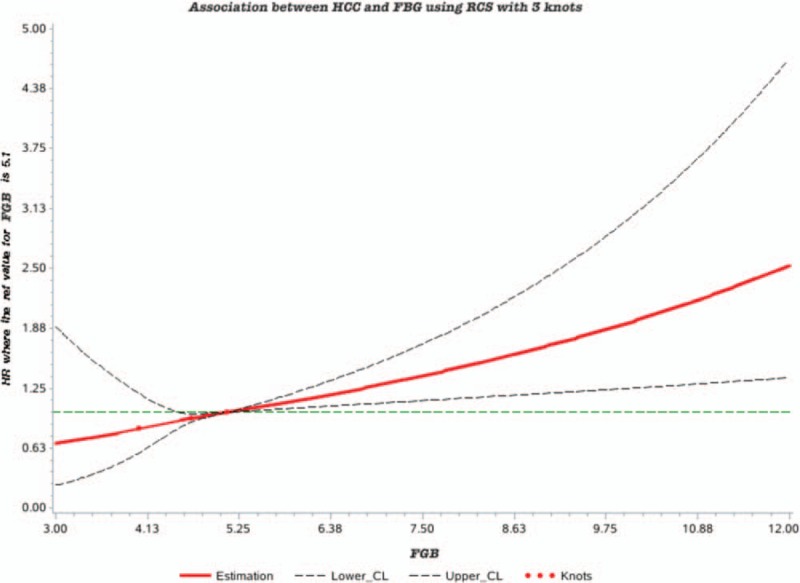
Association between HCC and FBG using RCS with 3 knots. Cubic spline graph of the adjusted HR (represented by solid line) and 95% CI (represented by the dotted lines) for the association between FBG and risk of HCC.

During the mean 8.67 ± 1.36 years of follow-up of 93,447 participants, 6624 individuals died before the occurrence of HCC. Table 3 summarizes crude and adjusted HRs (95%CI) for newly diagnosed HCC events after taking competing risk event (death) into the consideration. In cause-specific hazard model (CS model), the multivariable HRs (95%CI) for the association of FBG and FBG with HCC were 1.46 (1.09∼1.98), 1.69 (1.27∼2.27) in the multivariate adjusted analysis. Similar results were also observed in sub-distribution hazard function model (SD model) with corresponding multivariate HRs (95%CI) of 1.46 (1.09∼2.00), 1.69 (1.25∼2.27) in 4.82 mmol/L≤FBG≤5.49 mmol/L group and FBG >5.49 mmol/L group, respectively (Fig. 2).

### 3.5 Sensitivity analysis

After excluding participants with a history of diabetes in the baseline, similar results were also observed in both COX regression models and competing risk regression models, which were adjusted for the same potential confounders. In COX regression model, the multivariable adjusted HRs (95% CI) were 1.46 (1.08∼1.97), 1.60 (1.17∼2.19) in participants with FBG 4.82∼5.49 mmol/L and FBG >5.49 mmol/L. Almost the same results were obtained in CS models and SD models. Furthermore, 39 individuals who were diagnosed with HCC within 1 year entering the cohort were excluded in an analysis. There was still a positive association of the risk of HCC and elevated FBG in all models after eliminating the effect of major potential confounders (Supplement Table 1, Supplement Table 2).

## 4 Discussion

To our knowledge, there were few prospective studies focusing on the relationship between circulating levels of FBG and the risk of HCC. In this large prospective cohort study among 93,447 Chinese participants, one found that elevated FBG concentrations were significantly associated with an increased risk of HCC in both continuous variable analyses and categorical analyses even adjusted for suspected confounders. Furthermore, FBG concentrations were nonlinearly related to HCC risk, and the adjusted HRs of HCC related to FBG levels rose steadily among target participants. The main findings were not altered after excluding participants with diabetes or diagnosed with HCC within 1 year.

In this large prospective study, participants with 4.82 mmol/L≤FBG≤5.49 mmol/L and FBG >5.49 mmol/L had 47% and 69% increased risk of HCC versus the lowest FBG level. Those findings were in line with observations in the exiting prospective cohort studies. Fujino et al^[16]^ reported a statistically significant positive association between diabetes and HCC (HR = 2.8, 95% CI: 1.5–4.9) among the general population in Fukuoka, Japan. A study conducted in the United States found persons hospitalized with diabetes and no known liver disease could expect a 2-fold increase in the risk of HCC compared with those without diabetes.^[17]^ A study conducted in Korea demonstrated a strong association between high levels of FBG and risk of liver cancer in males (HR = 1.7, 95% CI: 1.5–1.8) and females (HR = 1.2, 95% CI: 1.0–1.4) after adjustments were made for age, smoking, and alcohol use.^[18]^ However, studies concerning 578,700 Europeans and 2903 male Taiwanese failed to find such a relationship.^[19,20]^ Even an inverse relationship between FBG and HCC had been reported in 2 studies, neither of which reached statistical significance though.^[21,22]^ There was a controversy for diabetes being a risk factor for HCC. Diabetes was considered as a complication of cirrhosis and whether diabetes-stimulated HCC independent of cirrhosis remains uncertain.^[23]^ A meta-analysis involving 42 case–control and cohort studies showed a 2-fold increased risk of HCC associated with diabetes after adjustments were made for alcohol and viral hepatitis. However, similar results were not obtained in cirrhosis-adjusted studies.^[24]^ The results in this study suggested that elevated FBG was a risk factor of new-onset HCC, independent of cirrhosis or other liver disease (NASH/NAFLD, alcoholic liver disease).

Prognostic models that estimate the actual individual risk were required to be as accurately as possible. Traditional multivariate COX regression may substantially overestimate the absolute risk of the event of interest because subjects with a competing event are treated as if they could experience the event of interest in the future especially in frail or elderly populations.^[25]^ In this study, 6624 death cases precluded the event of interest (HCC) and thus the benefit of an intervention, prognostic models should take competing risk events into account. A positive association between high levels of FBG and risk of HCC was found in both SD models and CS models. Former literatures have proved that the cause-specific hazard ratio and sub-distribution hazard ratio are distinct, and the choice of approach should be driven by the scientific question. The CS model might be more applicable for studying the etiology of diseases, whereas the SD model might be more appropriate for predicting an individual's risk for an outcome or resource allocation.^[26]^

The mechanisms that elevated FBG increased the risk of HCC remain uncertain. Several possible mechanisms might explain the association. Hyperinsulinemia leads to increased expression of insulin-like growth factor (IGF)-I expression, which is associated with tumor growth in vitro, in animal models, and in epidemiological studies in humans.^[27]^ Basic research found glial cell line derived neurotrophic factor (GDNF) and its tyrosine kinase receptor RET expression in BxPC-3 and MIA PaCa-2 cells when exposed to different concentrations of glucose.^[28]^ High glucose concentration was capable of accelerating tumorigenesis in humans. Several studies have demonstrated that diabetes is a disease that increases in the level of DNA damage, and the level of damage increases sharply with the loss of glycemic control.^[29,30]^ In addition, a previous study proved that glucose-induced signal triggering the disassembly of quiescent cells of specific structures depends on glucose catabolism through glycolysis.^[28]^ Also, high glucose condition could promote the proliferation and metastatic potential of cancer cells.^[31]^

Males demonstrated higher incidences than females in this study, which was in line with a previous study that males are almost 4-fold as likely as females to develop HCC.^[5]^ Men are known to have higher risk of HCC than women in several studies.^[32,33]^ Basic researches have demonstrated both protective effects of estrogens and deleterious effects of androgens contribute to the sexual dimorphism in HCC incidence.^[34,35]^

The current study is a large-scale community-based study, with a good number of incident HCC cases allowing for the full consideration of the most important risk factors of HCC. The strength of this study also includes the prospective design, which is better suited to examine the temporal association between potential exposure and the disease and is less subjected to recall bias. In addition, another strength is the almost 100% follow-up rate among the target population via biennially follow-up examination, and comprehensive health system, including death certificates, medical records, and health insurance. Moreover, the broad assessment of potential confounders, including age, sex, BMI, ALT, cirrhosis, HBV infection, NASH/NAFLD, alcoholic liver disease, current smoker, drinking status, physical activity had been well addressed in this study.

There are several limitations that should be noticed in our study. First, the mean follow-up time was 8.67 years, which is relatively short and may not have been long enough to detect a true relationship between FBG and the risk of HCC. Second, no data concerning HCV infection could be used in our study. However, HCV had much less effect on the development of HCC in Chinese than in other Asian population.^[36]^ Third, because of the industrial nature of Kailuan Community, there was an imbalance in sex distribution, with more men than women. But the bias concerning sex distribution on the results can be minimized as regression modes were adjusted for sex.

In summary, this prospective cohort study showed that higher FBG concentrations rather than diabetes were positively associated with new-onset HCC even in the competing risk models. Vaccination and treatment for HBV infection and newborns that began in China in the mid-1980s will certainly lead to diminished rates of HCC. But the vaccinated population is only in their 30s at the present time and contributed little to the current HCC incidence. On the basis of this large population-based study, FBG concentrations can be used as a scientific and important way to identify individuals with a higher risk of HCC and well control of FBG concentrations might serve as a possible way to decrease the risk of HCC among Chinese population.

## Acknowledgment

We thank the staff and participants of the Kailuan study for their important contributions.

## Author contributions

**Formal analysis:** Tong Liu, Wanchao Wang, Yiming Wang.

**Methodology:** Xining Liu, liying cao.

**Software:** Haozhe Cui, Miaomiao Sun, Yiming Wang.

**Writing – original draft:** Tong Liu, Hai Liu.

**Writing – review & editing:** Siqing Liu.

## Supplementary Material

Supplemental Digital Content
