# Supplementary material for: Elevated fasting serum glucose levels increase the risk of hepatocellular carcinoma: A prospective cohort study
Source: Medicine (Baltimore). 2019 Jul 26;98(30):e16369. doi: 10.1097/MD.0000000000016369 (PMC6709261; doi:10.1097/MD.0000000000016369)
Supplement: Supplemental Digital Content [file medi-98-e16369-s001.doc]

**Supplement Figure**


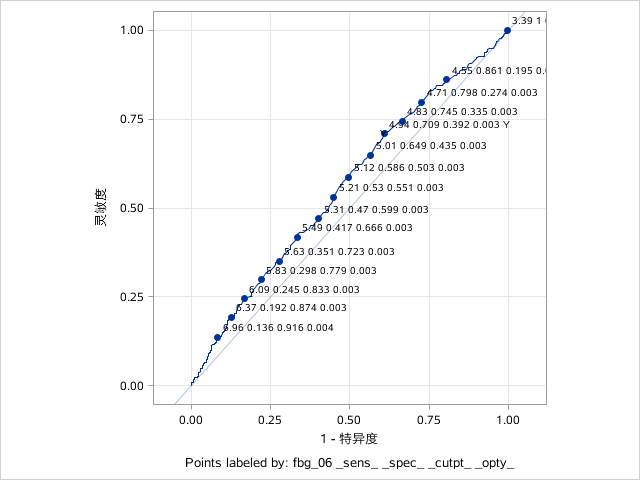


Table S1. Hazard ratios and 95%confidence interval(CI) for risk of HCC among participants stratified by FBG subgroups in different regression models excluding participants with history of diabetes in the baseline.

|  |  | **Fbg** | | | |
| --- | --- | --- | --- | --- | --- |
|  |  | **<4.82 mmol/L** | **4.82-5.49mmol/L** | **>5.49 mmol/L** | ***P* for trend** |
| **Multivariate COX Regression** |  |  |  |  |  |
|  | **Cases** | **76** | **100** | **85** |  |
|  | **Person-years** | **268209** | **271900** | **199371** |  |
|  | **Model 1** | **1.00(Ref.)** | **1.30(0.96~1.75)** | **1.51(1.11~2.05)** | **0.0324** |
|  | **Model 2** | **1.00(Ref.)** | **1.33(0.99~1.80)** | **1.41(1.04~1.93)** | **0.0667** |
|  | **Model 3** | **1.00(Ref.)** | **1.46(1.08~1.97)** | **1.60(1.17~2.19)** | **0.0082** |
| **CS Model** |  |  |  |  |  |
|  | **Cases** | **76** | **100** | **85** |  |
|  | **Person-years** | **268209** | **271900** | **199371** |  |
|  | **Model 1** | **1.00(Ref.)** | **1.29(0.95~1.73)** | **1.50(1.10~2.04)** | **0.0219** |
|  | **Model 2** | **1.00(Ref.)** | **1.32(0.97~1.75)** | **1.40(1.03~1.92)** | **0.0660** |
|  | **Model 3** | **1.00(Ref.)** | **1.46(1.17~2.19)** | **1.60(1.17~2.19)** | **0.0077** |
| **SD Model** |  |  |  |  |  |
|  | **Cases** | **76** | **100** | **85** |  |
|  | **Person-years** | **268209** | **271900** | **199371** |  |
|  | **Model 1** | **1.00(Ref.)** | **1.30(0.96~1.75)** | **1.50(1.10~2.05)** | **0.0328** |
|  | **Model 2** | **1.00(Ref.)** | **1.33(0.99~1.80)** | **1.41(1.03~1.94)** | **0.0675** |
|  | **Model 3** | **1.00(Ref.)** | **1.46(1.08~1.99)** | **1.60(1.17~2.22)** | **0.0081** |

Note: Model 1: Univariate analysis.

Model 2: Adjusted for age, sex based on model 1.

Model 3: Further adjusted for BMI, ALT, cirrhosis, hepatitis B virus infection, NASH/NAFLD, alcoholic liver disease, current smoker, drinking status, hypertension，physical activity based on model 2.

CS model: In cause-specific hazard model; SD: sub-distribution hazard function model.

**Table S2. Hazard ratios and 95%confidence interval(CI) for risk of HCC among participants stratified by FBG subgroups in diff**erent regression models excluding participants who occurred HCC within 1 year.

|  |  | **Fbg** | | | |
| --- | --- | --- | --- | --- | --- |
|  |  | **<4.82 mmol/L** | **4.82-5.49mmol/L** | **>5.49 mmol/L** | ***P* for trend** |
| **Multivariate COX Regression** |  |  |  |  |  |
|  | **Cases** | **63** | **92** | **108** |  |
|  | **Person-years** | **269079** | **272784** | **268618** |  |
|  | **Model 1** | **1.00(Ref.)** | **1.45(1.05~2.00)** | **1.72(1.26~2.35)** | **0.0028** |
|  | **Model 2** | **1.00(Ref.)** | **1.49(1.08~2.05)** | **1.58(1.16~2.16)** | **0.0110** |
|  | **Model 3** | **1.00(Ref.)** | **1.63(1.18~2.26)** | **1.76(1.28~2.41)** | **0.0013** |
| **CS Model** |  |  |  |  |  |
|  | **Cases** | **63** | **92** | **108** |  |
|  | **Person-years** | **269079** | **272784** | **268618** |  |
|  | **Model 1** | **1.00(Ref.)** | **1.45(1.05~1.99)** | **1.72(1.26~2.35)** | **0.0028** |
|  | **Model 2** | **1.00(Ref.)** | **1.49(1.08~2.05)** | **1.58(1.15~2.16)** | **0.0110** |
|  | **Model 3** | **1.00(Ref.)** | **1.63(1.18~2.25)** | **1.75(1.28~2.41)** | **0.0012** |
| **SD Model** |  |  |  |  |  |
|  | **Cases** | **63** | **92** | **108** |  |
|  | **Person-years** | **269079** | **272784** | **268618** |  |
|  | **Model 1** | **1.00(Ref.)** | **1.45(1.05~2.00)** | **1.70(1.25~2.32)** | **0.0034** |
|  | **Model 2** | **1.00(Ref.)** | **1.48(1.07~2.05)** | **1.57(1.14~2.15)** | **0.0131** |
|  | **Model 3** | **1.00(Ref.)** | **1.63(1.18~2.27)** | **1.74(1.26~2.41)** | **0.0018** |

Note: Model 1: Univariate analysis.

Model 2: Adjusted for age, sex based on model 1.

Model 3: Further adjusted for BMI, ALT, cirrhosis, hepatitis B virus infection, NASH/NAFLD, alcoholic liver disease, current smoker, drinking status, hypertension，physical activity based on model 2.

CS model: In cause-specific hazard model; SD: sub-distribution hazard function model.
